# Cauda Equina Syndrome Secondary to Incidental Hemorrhagic Ependymoma Following Spinal Anesthesia: A Case Report and Comprehensive Review

**DOI:** 10.7759/cureus.40490

**Published:** 2023-06-15

**Authors:** Ramy Samargandi, Abdulrahman Alesawi, Louis-Romée Le Nail, Osamah Abualross

**Affiliations:** 1 Department of Orthopedic Surgery and Traumatology, Centre Hospitalier Régional Universitaire (CHRU) de Tours, Tours, FRA; 2 Department of Orthopedic Surgery, Faculty of Medicine, University of Jeddah, Jeddah, SAU; 3 Faculty of Medicine, University of Jeddah, Jeddah, SAU

**Keywords:** incidental, case report, spinal anesthesia, ependymoma, hematoma

## Abstract

Spinal ependymomas are rare primary central nervous system tumors that often exhibit vague symptoms before being identified. In extremely rare situations, it can be identified after a neurological decline following a history of spinal anesthesia, indicating intraspinal hemorrhages from an incidental lumbar ependymoma that was not previously diagnosed. Spinal anesthesia is widely utilized in numerous orthopedic surgical procedures, as it is a well-tolerated invasive procedure with a low risk of complications. The patient in this case study underwent elective orthopedic surgery under general anesthesia following two unsuccessful trials with spinal anesthesia. Subsequently, the patient developed paraplegia as a result of an incidental hemorrhagic spinal ependymoma. The patient had an L3 laminectomy for decompression of the dural sheath, and an ependymoma was confirmed based on the histopathological assessment. This case report aims to raise awareness regarding the potential complication of spinal anesthesia arising from incidental tumors of the spinal cord, thereby emphasizing the imperative of early recognition and management in order to mitigate adverse outcomes.

## Introduction

Ependymoma is a rare primary tumor that accounts for approximately 2% of all primary central nervous system tumors. When present, it is located 75% of the time within the spinal cord [1]. Patients with spinal cord ependymoma (SCE) often complain of progressive nonspecific symptoms before being diagnosed, while there are rare occasions where intratumoral bleeding can cause abrupt worsening [2]. Neuraxial anesthesia is commonly performed for orthopedic surgeries involving the lower limb. Spinal anesthesia is a type of neuraxial anesthesia generally regarded as a well-tolerated invasive procedure with a low risk of complications [3].

Several studies have reported intraspinal hematomas with neurological decline after spinal needle insertion, indicating intraspinal hemorrhages from an incidental lumbar ependymoma that was not previously diagnosed before spinal anesthesia [4-13]. Herein, we present a case of a patient who underwent elective orthopedic surgery under general anesthesia after a failed attempt at spinal anesthesia and afterward experienced cauda equina syndrome due to an incidental hemorrhagic spinal ependymoma. Additionally, we look into the literature on incidental hemorrhagic spinal cord ependymomas that present with an acute neurological deficit as a complication after spinal anesthesia and address the best course of management for recuperation from this neurological impairment.

## Case presentation

A 44-year-old medically free female patient was admitted to the orthopedic department for a curettage biopsy of a cartilaginous lesion located in the right proximal tibia. The lesion was identified through radiological imaging after the patient reported experiencing knee pain persisting for a duration of one year. She underwent curettage biopsy and grafting under general anesthesia due to two failed trials of spinal anesthesia. The procedure was successful and without difficulties, showing no manifestation of neurological impairment, and the patient was discharged on the same day of surgery with a prescription for pain medication and anticoagulants. Five days later, the patient started to have lower back pain over the sacral area and lower limb weakness. On day 9 postoperatively, she was admitted for acute low back pain associated with sensory-motor deficit. Examination revealed diminished reflexes and sensory-motor deficit, lower back pain severely increasing on mobilization, and inability to be in the supine position. The neurological examination found lower limb deficit, and muscle power revealed the following: right ankle dorsiflexion: 1/5, left foot dorsiflexion: 1/5, right foot plantar flexion: 1/5, and left foot plantar flexion: 2/5. Quadriceps examination was limited due to knee pain, and ankle reflexes were diminished. Sensory examination revealed non-systematic hypoesthesia involving lower leg and foot reflex and no anesthesia in the saddle. The patient also presented with urinary retention, and a Foley catheter was inserted. A digital rectal examination revealed no sphincter abnormality. Clinical examination and a history of two failed spinal anesthesia procedures have raised suspicion of a post-spinal anesthesia-related compressive hematoma.

**Figure 1:**
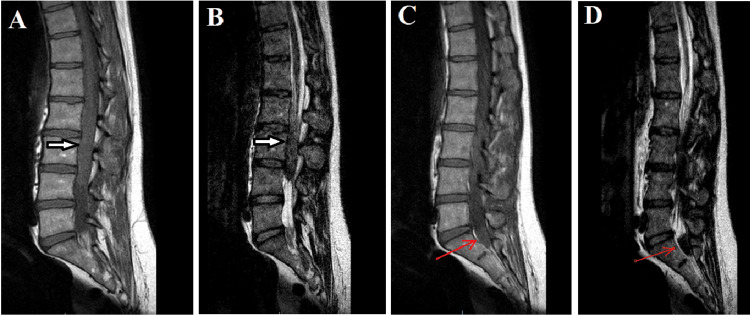
Preoperative MRI demonstrating lesions A: Sagittal T1-weighted MRI of the first lesion (white arrow). B: Sagittal T2-weighted MRI of the first lesion (white arrow). C: Sagittal T1-weighted MRI of the second lesion (red arrow). D: Sagittal T2-weighted MRI of the second lesion (red arrow). MRI: magnetic resonance imaging

**Figure 2:**
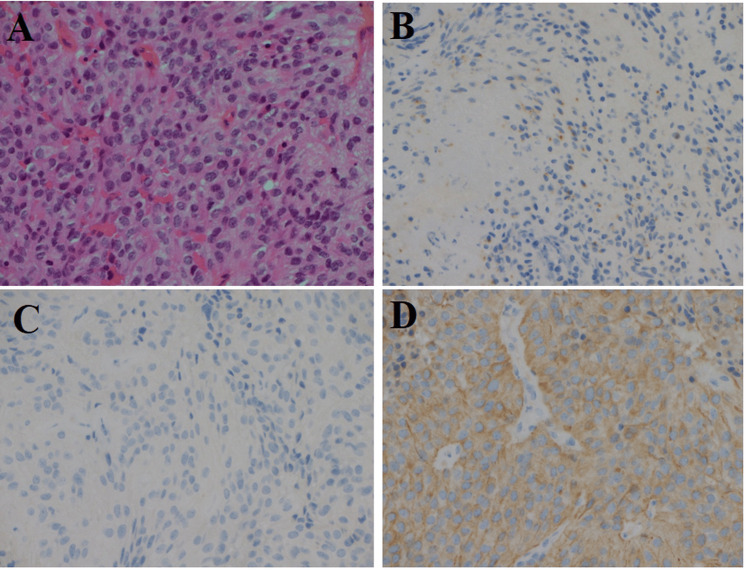
Histopathological examination of the lesion with different stains revealing the presence of atypical oval nuclei in the tumor, which indicates a grade II ependymoma according to the WHO classification A: Hematoxylin and eosin staining (magnification: ×20). B: PS100 staining. C: GFAP staining. D: EMA staining. WHO: World Health Organization, GFAP: glial fibrillary acidic protein, EMA: epithelial membrane antigen, PS100: S100 protein

An immediate spinal magnetic resonance imaging (MRI) indicated a possible post-spinal anesthesia hematoma, adjacent to L2-L4 (Figure 1). Considering the symptoms, emergent neurosurgery referral and surgery were indicated for possible cauda equina syndrome. The patient underwent L3 laminectomy for decompression of the dural sheath. Following the incision of the dura mater, a significant hematoma associated with a soft tissue lesion was discovered. The intradural hematoma was evacuated, and the soft tissue mass was excised and subsequently sent for pathological evaluation. Based on the findings of the pathology report, the soft tissue lesion exhibited histological characteristics consistent with an ependymoma (Figure 2).

**Figure 3:**
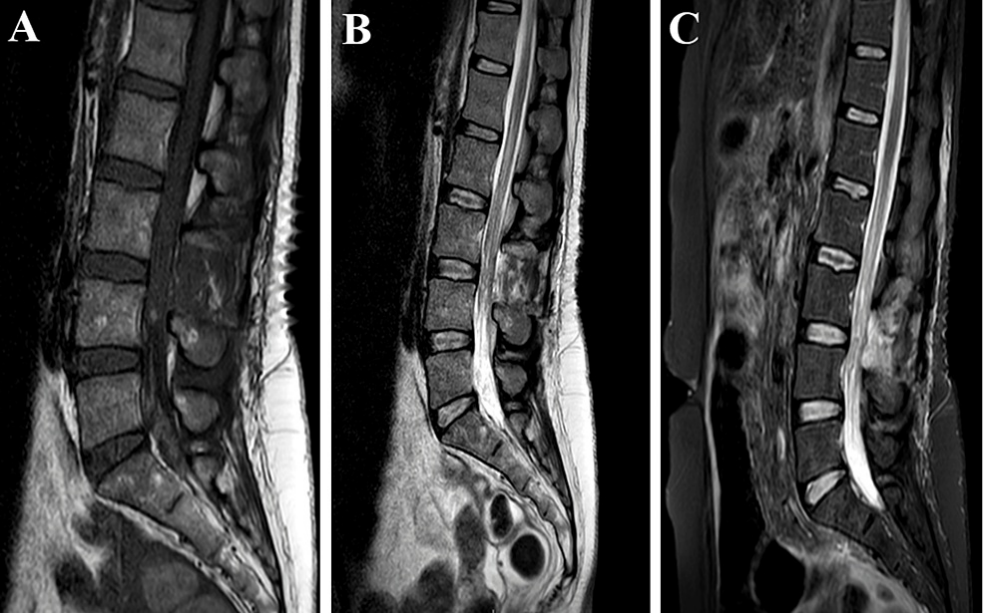
Immediate postoperative MRI after the decompression and removal of the lesion A: Sagittal T1-weighted MRI of the spine. B: Sagittal T2-weighted MRI of the spine. C: Sagittal STIR MRI of the spine. MRI: magnetic resonance imaging, STIR: short-TI inversion recovery

Postoperatively, there was partial recovery of the right lower limb (extension: 3/5), no recovery of the left foot (extension: 2/5), reduction of lower back pain, and absence of anesthesia in the saddle. MRI confirmed good decompression of the roots of the cauda equina, but there was an image at the level of the dural sac strongly suggestive of a second tumor location (Figure 3). Physiotherapy was initiated as soon as possible for the rehabilitation of deficits. Two days postoperatively, preventive anticoagulant was resumed. The case had been discussed in a tumor board, and simple monitoring by MRI had been proposed, the first at three months and every six months for two years.

**Figure 4:**
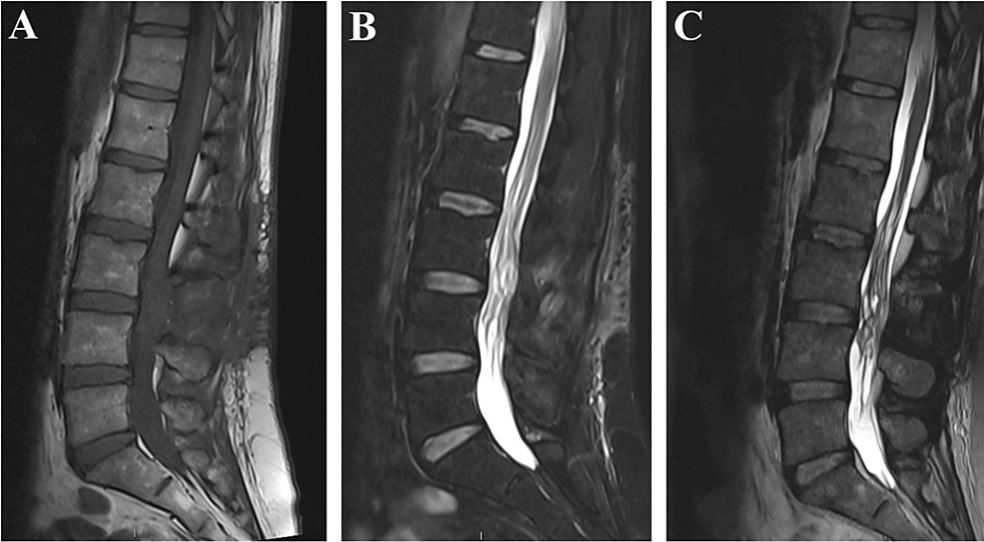
Three-month postoperative MRI A: Sagittal T1-weighted MRI of the spine. B: Sagittal T2-weighted Dixon water MRI of the spine. C: Sagittal three-dimensional SPACE T2 STIR MRI. MRI: magnetic resonance imaging, STIR: short-TI inversion recovery, SPACE: sampling perfection with application-optimized contrasts using different flip angle evolution

The patient received psychological support both after the operation and throughout the rehabilitation process. Three months after the intervention, the patient is in marked improvement on the neurological level. She walked with two canes, and steppage is present, especially on the right. Sphincter function returned to normal, and the urinary catheter was removed. The patient does not complain of any pain and continues her rehabilitation through physiotherapy on a multi-weekly basis. The MRI performed three months postoperatively confirmed the perfect decompression of the roots of the cauda equina but above all, unexpectedly, the complete disappearance of the second lesion, which existed at the level of the dural sac (Figure 4). It is probably a hemorrhagic lesion that has therefore been resolved over time. In L3 and L4, the roots of the cauda equina seem to have recovered their place well.

Six months postoperatively, a remarkable improvement in neurological function was observed, with patients now walking without canes and returning to work part-time as a sales manager. However, there is still a residual deficit in muscle strength, rated at 4 out of 5. Follow-up is planned for at least two years, with MRI every six months, to assess the risk of recurrence and evaluate the neurological recuperation progress clinically.

## Discussion

The prevalence of incidental ependymoma and spinal anesthesia complications are rare in their own right, yet they have been described in previous literature. This case adds to the small pool of literature on this bizarre couple. As most of the previous cases described are related to obstetric procedures, this article presents a uniqueness as one of only two articles following an orthopedic procedure [6]. In addition, the reports ranged from less than 24 hours for the onset of symptoms [4-8] to, in some cases, including this, six months after surgery, which raises the possibility that such presentations of lower back pain and lower limb paraplegia can indeed present as late findings of spinal anesthesia-induced hemorrhagic ependymoma [9-12]. Accordingly, a history of paraplegia after a successful or failed attempt at spinal/epidural anesthesia should raise suspicion for possible ependymoma, given the propensity of ependymomas to hemorrhage due to their vascular structures [4]. The typical level of the lesion above L3 was mentioned in several instances [5,8,10,11,13]. However, despite being unusual, ependymomas have been located within and below L3, which is the level of puncture for most spinal anesthesia [4,6,7,9,12]. It is also worthwhile to mention the possibility of metastatic spinal lesions as a potential culprit, as similar cases have been described where patients suffer comparable outcomes post-neuraxial anesthesia due to undiagnosed metastatic cancer (Table 1) [14].

**Table 1:** Reported cases of hemorrhagic spinal ependymoma presenting with acute neurological deficit post-spinal anesthesia MRI: magnetic resonance imaging

Author (year)	Patient’s age and gender	Indication of neuraxial anesthesia	Neurological manifestations	Onset of signs and symptoms	Tumor (location)	Treatment (timing after presentation)	Prognosis
Roscoe et al. (1984) [9]	24, female	Cesarean section	Low back pain, numbness/weakness of the left leg	4 days	Ependymoma (L1-L4)	L1-L4 laminectomy (1 day)	Residual motor weakness
Antoniadis et al. (1985) [6]	79, female	Removal of femoral osteosynthesis	Weakness in both legs, urinary incontinence	Immediately postoperatively	Ependymoma (L1-L5)	L1-L5 laminectomy (25 days)	Improving motor and bladder function
Martin et al. (1992) [7]	31, female	Cesarean section	Low back pain	Immediately postoperatively	Ependymoma (L4-L5)	L4-L5 laminectomy (6 months)	Full recovery
Jaeger et al. (2002) [8]	24, female	Cesarean section	Low back pain, paresthesia, complete paraplegia	12 hours	Ependymoma (T12-L2)	T12-L2 laminectomy (8 hours)	Improving motor function, absent bladder function
Campbell et al. (2008) [10]	27, female	Cesarean section	Low back pain with bilateral radiculopathy	3 days	Ependymoma (T12-L2)	T12-L2 laminectomy (3 weeks)	Full recovery
Armstrong et al. (2010) [13]	24, female	Cesarean section	Asymptomatic, incidental finding on MRI	Asymptomatic	Ependymoma (L2-L3)	Surgical resection of the tumor (delayed 3 months)	Completely recovered
Fournet-Fayard et al. (2012) [11]	31, female	Vaginal delivery	Paresthesia (right side)	3 months	Ependymoma (T1-T6)	Surgical resection of the tumor (after 21 months)	Unrecovered, complete paraplegia due to extensive spinal cord atrophy
Rosario et al. (2015) [4]	22, male	Urological procedure, failed lumbar puncture	Bilateral lower limb weakness and numbness, bladder, and bowel incontinence	Post-procedure progressed for 6 months	Ependymoma (L2-S2)	Posterior decompression, tumor resection (6 months)	Improving but absent bladder and bowel function
Lee et al. (2016) [5]	24, female	Cesarean section	Paraplegia	Immediately after delivery	Ependymoma (C2-T5)	Left C6-T2 laminectomy (2 weeks)	Residual motor deficit and cauda equina syndrome
Quintana et al. (2021) [12]	40, female	Cesarean section	Low back pain, bilateral numbness, urinary retention, difficult ambulation	6 days	Ependymoma (T11-L5)	T11-L5 laminectomy (13 days)	Residual motor weakness
Present report (2023)	44, female	Curettage and bone graft of a proximal tibial lesion	Low back pain, bilateral lower limb weakness, and urinary retention	5 days	Ependymoma (L3-L5)	L3-L5 laminectomy (4 days)	Residual motor weakness

The aim of surgery is to perform a wide resection with negative margins, as it is with the majority of other soft tissue tumors. The extent of excision is determined by the tumor’s size, histology, location, and whether or not it has a capsule or syrinx [2]. Due to the region’s relatively poor blood supply and constrained spinal canal, patients with thoracic-level tumors frequently experience higher functional losses following resection, which is supported by previous reports [2,4-13]. As demonstrated, almost all patients showed some form of motor sensory recuperation [4-13]. However, patients who have maintained neurological function prior to surgery are less likely to experience postoperative neurological symptoms than those who have deficits at baseline and are more likely to recover from those symptoms when they do [2]. Moreover, it has been shown that even late surgical evacuation and excision can still aid in the improvement of the patient’s condition as demonstrated in previous cases, along with prolonged courses of physiotherapy and/or radiotherapy in hopes of improving function and reaching better outcomes [4,6,7,10]. When it comes to prevention, we cannot fully recommend the use of radiological imaging before every neuraxial anesthesia attempts to avoid such cases, due to poor cost-effectiveness. It is worth mentioning, as others have, the necessity of careful evaluation for spinal mass symptoms prior to any invasive spine procedure. Through careful history-taking and thorough examination, the choice of neuraxial anesthesia can be substituted in some patients if patient risks are suspected while also not neglecting the immediate evaluation of new or worsening post-procedural neurological symptoms.

## Conclusions

Although such incidences are deemed to be rare, this case demonstrates the importance of vigilance when it comes to postoperative neurological manifestations concerning invasive spinal procedures. While patients from this and the abovementioned reports showed favorable improvement with subsequent treatment even with what’s considered a later presentation, it is still advisable to act as quickly as possible with these cases to ensure the best possible result. Efforts to educate patients to seek faster care could help in reaching better outcomes.
